# Associations between body composition and fundamental motor skill competency in children

**DOI:** 10.1186/s12887-021-02912-9

**Published:** 2021-10-11

**Authors:** E. Kipling Webster, Indica Sur, Alicia Stevens, Leah E. Robinson

**Affiliations:** 1 Institute of Public and Preventive Health, Augusta University, 1120 15th Street, Augusta, GA 30912 USA; 2 School of Kinesiology, University of Michigan, 830 North University, Ann Arbor, MI 48109-1048 USA

**Keywords:** BIA, BMI, Pediatrics, Motor skills, Obesity, Body composition

## Abstract

**Background:**

Evidence supports an inverse relationship between weight status and motor competence, but most work utilizes body mass index as the proxy for weight status. Body mass index fails to account for essential components of body composition, which may be critical for motor performance. The purpose of this investigation was to examine the relationship between fundamental motor skills competency and body composition (i.e., fat mass, fat percentage, and fatfree mass) as measured by bio-electrical impedance analysis and body mass index in children.

**Methods:**

Two hundred forty-four children from the Southeastern portion of the United States participated in this project (6.05 ± 2.01 years, 53.3% male). Fundamental motor skills were measured using the Test of Gross Motor Development – 2nd edition and body composition was assessed with the Tanita SC-331S Body Composition Analyzer (bio-electrical impedance analysis). Body mass index was calculated using CDC normative growth charts.

**Results:**

Bio-electrical impedance analysis measures accounted for 23.1%, F(3, 241) = 24.10, *p* < .001 and 2.7%, F(3, 241) = 2.22, *p* = .086 variance in locomotor and object control subscales, respectively; body mass index accounted for 8.4% (locomotor) and 0.1% (object control) variance. For the Test of Gross Motor Development -2nd edition total score, bio-electrical impedance analysis measures accounted for 24.4% F(3, 241) = 25.90, *p* < .001 compared to body mass index which accounted for 7.9% F(1, 244) = 20.86, *p* < .001 of the variance. Only fat free mass (p < .001) was a significant predictor for locomotor skills and total models for the Test of Gross Motor Development – 2nd edition; BMI was also a significant predictor (*p* < .001) in both the locomotor and total models.

**Conclusions:**

Different components of body composition (i.e., fat free mass) were associated with different aspects of fundamental motor skills competency. Excess body fat may be a morphological constraint to proficient locomotor performance when transporting the body through space. In contrast, body composition did not significantly predict object manipulation performance. More work is needed to understand the causality and directionality of this relationship; however, bio-electrical impedance analysis accounts for more variance in fundamental motor skills performance than body mass index in a field-based setting.

In the United States, an estimated 19.3% of U.S. children (2–19 years) children are obese [1]. Childhood obesity is associated with negative health consequences later in life, including Type 2 diabetes [2], hypertension [3, 4], and high cholesterol [5]. Additionally, higher body mass index (BMI) during childhood predicts aspects of prediabetes and cardiovascular diseases in adulthood [6]. With the growing concerns related to childhood obesity, research has explored the role of modifiable factors (e.g., fitness levels, physical activity participation, and motor skill competence) that might contribute to healthy weight in children and youth [7–12]. Overall, these studies have found that low levels of fitness, motor competence, and physical activity are associated with a higher weight or BMI.

An abundance of research has examined the inverse relationship between motor competence and unhealthy weight in children [7–9, 13–16]. Cross-sectionally, higher BMI is associated with lower motor coordination [17, 18] and children with more inadequate motor coordination had a higher risk of being overweight [19]. Longitudinally, a child’s current weight status influences gross motor coordination later in life [9, 20]; specifically, children’s BMI at baseline predicted and explained 37.6% of the variance in gross motor coordination with age [9]. However, many of these studies utilize BMI as their indicator for overweight and obesity, which serves as a potential limitation to the generalization of these previous results.

In weight-related research, overfatness is what is measured to determine obesity status and BMI is the common metric used. BMI is calculated based on an individual’s weight and height. For children and adolescents, BMI percentiles are generated based on age and gender-specific normative data to characterize a child as underweight, normal weight, overweight, or obese [21, 22]. BMI is a widely accepted tool and proxy measure for assessing weight status [23], but it is only a surrogate (i.e., indirect) measure of body fatness [24]. BMI does not account for body composition (such as lean muscle mass, fat-free mass, or body fat percentage), nor does it examine body fat distribution [25], which have been shown to be an important risk factor for children’s health [26]. More importantly, Maynard et al. [27] found that body composition changes in children are generally attributed to lean rather than fat composition changes, which is difficult to determine solely with BMI. In addition, a meta-analysis recently showed that BMI calculations had 73% sensitivity in detecting obesity, meaning almost a quarter of children with obesity may go undetected [28]. There are other measures of body composition that examine body fat, both directly and indirectly, that may provide a more accurate picture in understanding the relationship between body fat and motor competence.

Body composition can be measured in children both in a lab or clinical-setting as well as in the field. Lab-based measures such as dual-energy X-ray absorptiometry (DXA) and air displacement plethysmography (Bod Pod) give accurate measures of bone mineral density, body composition, and body density but can be expensive, time-consuming, and less accessible. On the other hand, field-based measures are far more accessible and thus more readily used in larger studies or more frequently for screenings, but accurate estimates are dependent on the accuracy of the data collector and limitations of the measurement technique itself [29]. Bioelectrical impedance analysis (BIA) collects accurate body composition measurements in children [30, 31]. Additionally, BIA is portable, objective, making it a more plausible option for use in the field. Thus, a more detailed, field-based measurement of body composition might shed new light on the association of weight status and motor competence in children.

Several studies have found an inverse association between fundamental motor skill (FMS) competency and obesity, as measured by body composition. Henrique et al. [32] found central obesity (as measured by waist-to-height ratio) was negatively associated with locomotor skill performance in preschool-age children. Lopes et al. [33] found that body fat percentage and waist circumference were negatively related to motor competence in older girls. In contrast, poor motor competence was associated with BMI, waist circumference, waist-to-height ratio, and body fat percentage in boys. Slotte et al. [34] concluded there is an inverse associate between FMS and body composition as measured with DXA, specifically, higher FMS scores were significantly and strongly associated with a lower body fat percentage and lower abdominal region fat percentage in 8-year old children. To date, only one study has utilized BIA compared to motor competence in young children (i.e., 5–6 years of age). They found higher body fat percentage in girls were negatively associated with strength and agility (as measured by the BOTMP), no associations were found for boys [35]. More work is needed to explore body composition more holistically in children in relation to performance of FMS.

Given the limitations associated with BMI as a measure of obesity, additional evidence is needed to assess the relationship between motor skill competence and weight status in children. There is a need for more research that incorporate additional measures of body composition (e.g., BIA) in order to understand this association in greater detail. The purpose of this study is to examine the associations between FMS and body composition using BIA in children. A secondary aim is to explore the association FMS and BMI, to compare results with the measurements taken from the BIA.

## Methods

The present study consisted of a convenience sample of children from one preschool and one elementary school in the Southeastern region of the United States. All children from both schools in grades preschool through 3rd grade were invited to participate in the current project, and 283 children returned an informed consent document. Thirty-nine children were removed from the final analysis due to incomplete data sets (incomplete motor skill data *n* = 12, incomplete weight data *n* = 25, absent on all measurement dates/no data n = 2). The current study design and procedures received Institutional Review Board approval and oversight, and both written parental consent and verbal child assent were obtained before data collection.

### Body composition

Height was measured using a portable stadiometer (nearest 0.1 cm), and weight was measured using a digital SECA scale (nearest 0.1 lb.; SECA GmbH & Co. KG, Hamburg, Germany). BMI and BMI percentiles were calculated from height and weight measurements using the U.S. Centers for Disease Control and Prevention (CDC) age and sex-specific growth charts [23]. Children who have a BMI percentile under the 5th percentile are considered underweight, between the 5th and 85th percentile are considered normal weight, over the 85th percentile are considered overweight, and above the 95th percentile are considered obese [23].

Body composition was assessed using the Tanita SC-331S Body Composition Analyzer (Tanita Corporation, Tokyo, Japan). Height measurements, age, and sex were manually inserted into the BIA device. Per manual guidelines, children were assessed without shoes, socks, or heavy outer clothing. The BIA device assessed body composition through three outcome measures: fat mass (estimate of total body fat mass), fatfree mass (remaining non-fat body mass and a proxy of muscle mass) and fat percentage (proportion of fat mass to total body mass). The BIA device does have a recommended age limit of 5 years, however previous work has measured fat mass, fat free mass, and fat percentage in preschool age children (2–5 years) using a Tanita scale [36]. In children under the age of 5 years, the minimum age of the device was used (i.e., age 5) and research assistants ensured that each child’s foot contacted all electrodes prior to measurements. Using the fat percentage measurements, body fat reference curves [37] for children 5 years of age and older were used to classify children into percentiles. Children under the 2nd percentile were considered underfat, between the 2nd and 85th percentile were considered normal weight, above the 85th percentile were considered overfat, and above the 95th percentile were considered obese.

### Fundamental motor skill competency

FMS competency was assessed using the valid and reliable Test of Gross Motor Development – 2nd edition (TGMD-2) [38]. The TGMD-2 is a systematic observation protocol that measures gross motor skill performance in children between 3 and 10 years of age with both process- and product-oriented components. The TGMD-2 is comprised of 12 skills divided into two subscales, (1) locomotor skills: run, gallop, hop, leap, jump, slide; and (2) object control skills: two-hand strike, dribble, kick, catch, overhand throw, underhand roll. The data for this project was collected in 2013 before the TGMD-3 was available for use.

The TGMD-2 was assessed in small groups of 2–4 participants during the school day in an indoor gym and administered by a trained researcher according to assessment protocols. Participants observed a live demonstration of each skill before performing a practice trial followed by two scored trials. Performance of each skill was scored using 3–5 individual criteria (1 = criterion met, 0 = criterion not demonstrated); the criterion scores from both trials were summed to calculate a raw score for each skill. Skill scores are summed to create locomotor and object control skill subscale totals, and overall TGMD-2 total raw scores (range 0 to 96). Based on the child’s age and sex, raw scores were converted to percentile scores for our analyses. All data were video recorded and analyzed by trained research assistants who had established at least 90% inter-rater reliability with an expert motor development researcher; a second coder coded 25% of the data to ensure appropriate reliability.

### Statistical analysis

Six separate models were run, including the TGMD-2 subscales, both (1) locomotor skills and (2) object control skills, as well as (3) the total TGMD-2 scores for both the BIA and BMI measurements. Multiple linear regressions were used for all models to assess how well the body composition variables derived from the BIA (fat mass, fat-free mass, and fat percentage) or BMI derived from the CDC normative growth charts could predict TGMD-2 scores. TGMD-2 percentile scores were used in all analyses.

## Results

The final sample included 244 participants. The average age was 6.05 ± 2.01 years (range 3–10; n_Age3_ = 31, n_Age4_ = 49, n_Age5_ = 21, n_Age6_ = 23, n_Age7_ = 40, n_Age8_ = 60, n_Age9_ = 16, n_Age10_ = 4), 53.5% were male, and most children were African American (71.7%; 16.8% Hispanic, 9.0% Caucasian, 1.6% Asian, and 0.8% Mixed Race). Across the sample, the average CDC-derived BMI was 17.53 ± 3.96 (M = 58.14 ± 33.34); over half of the sample was considered normal weight (< 5th percentile, > 85th percentile; 61.3%) and approximately one-fifth were considered obese (>95th percentile; 20.8%). Using the BIA measurements, the average Tanita-derived BMI was 16.90 ± 3.88. In children over the age of 5 years (*n* = 168), half of the sample would be considered normal weight (< 2nd percentile, >85th percentile; 45.8%), while approximately a quarter of this sample would be considered obese (>95th percentile; 24.4%) using the body fat reference curve percentiles [37]. In terms of performance on the TGMD-2, children’s performance was relatively low, scoring in the 12th percentile overall. See Table 1 for full information.

**Table 1:** Descriptive information

	Total	Boys	Girls
	*n* = 244	*n* = 130	*n* = 114
Age	6.05 (2.01)	6.09 (2.04)	6.00 (1.98)
Race (%)			
Caucasian	9.0%	10.0%	7.9%
African American	71.7%	66.2%	78.1%
Hispanic	16.8%	20.0%	13.2%
Asian	1.6%	2.3%	0.9%
Mixed Race	0.8%	1.5%	0.0%
Tanita Measures			
Fat mass (kg)	5.78 (4.90)	5.86 (5.17)	5.69 (4.59)
Fat free mass (kg)	19.69 (6.03)	20.14 (6.55)	19.17 (5.35)*
Fat percentage	20.53 (7.26)	20.40 (7.24)	20.68 (7.30)
BMI	16.90 (3.88)	17.04 (3.67)	16.75 (4.12)
Body Fat Percentile^1^	53.06 (37.40)	57.75 (37.80)	43.05 (38.09)
Underfat	17.3%	14.8%	17.1%
Normal weight	45.8%	39.8%	55.3%
Overfat	12.5%	17.0%	6.6%
Obese	24.4%	28.4%	21.1%
CDC-dervied measures			
BMI	17.34 (3.93)	17.60 (3.79)	17.46 (4.16)
BMI Percentile	58.14 (33.34)	61.04 (32.91)	59.00 (32.38)
Underweight	6.3%	5.5%	7.1%
Normal weight	61.3%	61.4%	61.1%
Overweight	11.7%	10.2%	13.3%
Obese	20.8%	22.8%	18.6%
TGMD-2 total			
Raw scores	47.67 (14.12)	51.61 (14.81)	44.61 (12.86)
Percentile scores	12.15 (16.43)	13.23 (17.58)	10.92 (15.00)*
Locomotor skills			
Raw Scores	21.6 (7.17)	22.69 (7.46)	21.17 (6.89)
Percentile scores	9.71 (13.62)	10.88 (14.63)	8.37 (12.31)*
Object control skills			
Raw scores	26.01 (8.50)	28.92 (8.80)	23.44 (7.40)*
Percentile Scores	11.88 (12.21)	12.28 (12.27)	11.41 (12.17)

Note: M (SD); ^1^Calculated from McCarthy et al. [37] for children over the age of 5 years (total *n* = 168, boys *n* = 88, girl *n* = 76); *p <.05 sex differences

### Regression results

The first analysis examined locomotor percentile scores for the TGMD-2 with fat mass, fat percentage, and fat-free mass as predictors. Fat mass, fat percentage, and fat free mass were entered, and the total variance explained was 23.1%, F(3, 241) = 24.10, *p* < .001. In the model only fat free mass (p < .001) contributed significantly. The final model also showed a one SD increase in fat free mass was associated with a .595 point decrease (*p* < .001) in TGMD-2 locomotor skill percentile scores.

The second analysis examined object control percentile scores for the TGMD-2 with fat mass, fat percentage, and fat free mass as predictors. The BIA variables were not correlated to the object control percentile scores, therefore when the BIA variables were entered, none of the variables contributed significantly to the model, fat free mass (*p* = .065), fat percentage (*p* = .661), fat mass (*p* = .455). In the regression model, total variance explained was 2.7%, F(3, 241) = 2.22, *p* = .086.

The third analysis examined the association between total TGMD-2 percentile scores with fat mass, fat percentage, and fat free mass as predictors. The BIA variables were entered, and the total variance explained was 24.4% F(3, 241) = 25.90, *p* < .001. The full model had a fat free mass (*p* < .001) as a significant predictor, with fat mass (*p* = .427), and fat percentage (*p* = .965) not contributing significantly. The final model showed fat free mass was associated with a .595 point decrease (*p* < .001) in total TGMD-2 percentile scores.

Finally, multiple linear regressions were run for locomotor, object control skill and TGMD-2 percentile scores separately with CDC BMI as the predictor. For locomotor skills, the total variance explained by the full model was 8.4% F(1, 244) = 22.29, *p* < .001). CDC BMI was a significant predictor (p < .001), and a one SD increase of BMI was associated with a .289 point decrease in locomotor percentile scores. For object control percentile scores, the total variance explained by the full model was 0.1% F(1, 244) = .136, *p* = .713). Similar to the BIA results, CDC BMI was not a significant predictor (p = .713) and was not correlated with object control percentile scores. For total TGMD-2 percentile scores, the total variance explained by the full model was 7.9% F(1, 244) = 20.86, *p* < .001. CDC BMI was a significant predictor (p < .001), and a one SD increase was associated with a .281 point decrease in total TGMD-2 percentile scores. See Table 2 for full regression results.

**Table 2:** Results of Regression Analysis

	β	*p*	*F*	*df*	*R2*	*p*
Locomotor Scores			24.10	3, 241	0.231	<.001
Fat Percentage	−.097	.504				
Fat Mass	.230	.224				
Fat Free Mass	−.595	<.001				
Object Control			2.22	3, 241	0.027	.086
Fat Percentage	−.072	.661				
Fat Mass	−.158	.455				
Fat Free Mass	.207	.065				
TGMD - 2			25.90	3, 241	0.244	<.001
Fat Percentage	−.006	.965				
Fat Mass	.148	.427				
Fat Free Mass	−.595	<.001				
Locomotor Scores			22.29	1, 244	0.084	<.001
CDC BMI	−.289	<.001				
Object Control			.136	1, 244	0.001	.713
CDC BMI	.024	.713				
TGMD - 2			20.86	1, 244	0.079	<.001
CDC BMI	−.281	<.001				

## Discussion

The current project examined FMS associations, assessed by the TGMD-2, and body composition measurements (specifically body fat, fat percentage, and fat free mass) using BIA in children. A secondary aim was to examine the associations between FMS and BMI, to compare the results to the measurements taken from the BIA. Results indicated that different aspects of body composition were significantly associated with results from the TGMD-2. Specifically, significant associations were found between fat free mass and (1) locomotor skills and (2) total TGMD-2 scores. For BMI results, similarly both locomotor skills and total TGMD-2 scores were significantly associated with BMI. Object control skills were not significantly associated with either BIA or BMI measures. In terms of variance explained for locomotor skills, BIA accounted for 23.1% of variance while BMI accounted for 8.4%. For object control skills, only 2.7% (BIA) and 0.1% (BMI) of variance was explained and no weight variables included in this project contributed to the final model. For total TGMD-2 scores, BIA measurements accounted for 24.4% of the total variance and BMI accounted for 7.9% variance in performance. These findings expand our current understanding of the relationship between body composition and FMS competency in children. The present study utilized a process-oriented FMS assessment which evaluates how proficiently a skill is performed [38]. This type of assessment and a more nuanced evaluation of body composition may provide critical insight when FMS are developing.

There is ample evidence showing that motor performance is negatively associated with higher weight in children [39, 40]. Lopes et al. [14] examined the effect of BMI on motor coordination in over 7000 children and adolescents. They found that children with low motor coordination (measured by a product-oriented assessment) had a higher risk of being overweight or obese. Excess weight can create a morphological constraint for children to move against gravity, resulting in less efficient movement patterns and more effort to displace body weight [41]. Constraints, which include those from the individual, task, and environment, can positively or negatively impact FMS development [42]. The negative constraints presented by excess weight can influence the proper execution of specific motor tasks, which may have a cascading impact on future movement behaviors, influencing future movement in more context-specific tasks [43]. Longitudinal work by D’Hondt et al. [40] further supports this assertion by providing evidence that over 2 years, children with obesity fall further behind their normal weight peers in motor coordination activities (measured by a product-oriented assessment). However, gaps still exist in the present literature based on the type of FMS used (product- vs. process-oriented assessments) and how weight is measured.

In the present study, locomotor skills had a negative association with body composition; children who had higher amounts of fat free mass tended to do worse on tasks like running, jumping, hopping, or leaping, when measured with a process-oriented assessment that examines how a skill is appropriately performed. Research related to physical activity and growth found that children who have higher fat free mass tend to have higher fat mass as well [44], which may explain why excess muscle mass in the present study may be detrimental to overall performance. These FMS require the transition of one’s entire body mass and/or center of gravity through space in a coordinated pattern [38, 43]. It is plausible that the rationale for the inverse relationship between motor skill competence and weight status is due to the body’s inability to execute propulsion and stabilization from increased weight in childhood. Therefore, increased fat free mass may negatively deter from proficiently completing these types of tasks until children are able to better to coordinate limbs in rhythmical and consistent movements as assessed by the process-oriented TGMD-2. Analyzing multiple components of weight through BIA allowed for more variance to be explained in performance on both the locomotor and total TGMD-2 which may provide nuanced ways of understanding the relationship between weight and FMS performance.

Utilizing BMI, previous work that used product-oriented assessments (i.e., an assessment which measures the outcome of a task) found that children who were obese tended to perform worse on jumping, balancing, and hopping tasks [13, 34, 45, 46]. Henrique et al. [32] found that preschool children who performed better on locomotor skills, using the same process-oriented assessment as the present study, tended to have lower levels of central obesity. Body fat percentage was also negatively associated with the TGMD-2 in 8-year-old children using the DXA [34]. The last results, which mirrors the present findings, highlight that a field-based measure may yield similar results furthering the current literature and practicality of replicating this work. The opportunity to examine more detailed body composition measures in a field-based setting may yield valuable, clinical information that could be used to screen more children, understand different components of weight more holistically, and allow for tracking of measurements over time to help understand growth more accurately.

The present study showed no association with body composition and motor skills that entail manipulating and projecting objects: object control skills. Previous work has also found that body composition (i.e., BMI and waist-to-height ratio) was not significantly associated with specific object control tasks in preschool children [13, 32, 45]. This lack of association may be because many object control tasks are stationary and do not require a complete displacement of body weight, but rather the spatial and temporal timing of limb movements. However, this lack of association may also be due to measurements, like BMI, not fully accounting for variance explained by different facets of body composition. Work by Matarma et al. [35] and Slotte et al. [34] found significant differences in body composition and object control skills using BIA and DXA, respectively. Matarma et al. [35] used BIA in young children and a product-oriented FMS assessment so no direct comparisons can be made to the skills measured from the present study; however, children who were overweight performed worse on body coordination and strength and agility tasks. In the other study, Slotte et al. [34] used the TGMD-2 to assess FMS and found that object control skills were significantly lower in overweight 8 year-olds compared to their normal weight peers by DXA measurement; however, this study found that fat free mass was not significantly associated with FMS performance. These mixed results from previous work and lack of associations found in the present study may indicate that the influence of body composition on object control skills may be dependent on the task itself, where some manipulation and projection of objects may not be influenced by excessive weight.

Total TGMD-2 scores were associated with fat free mass, likely reflecting the findings from the locomotor subscale. An interesting result was comparing the amount of variance the BIA measures and BMI measures account for in FMS performance. BIA measurements accounted for 24.4% of the variance, while BMI only accounted for 7.9%. One consideration is that the present study utilized a process-oriented FMS assessment, which may be more sensitive to subtle changes in performance based on morphological constraints compared to product-oriented assessments. Separating specific aspects of body composition may be imperative in addressing how morphological constraints may deter the acquisition of proficient movement patterns that contribute to FMS competency and how these can be overcome through modifications and continued practice. Previous work examining fat free mass and FMS in children is limited; further work may be needed using more nuanced body composition measurements to understand this finding in FMS fully.

An important consideration for the present study is that performance on the TGMD-2 in the present study was very low, children on average scored in the 12th percentile, and approximately one-fifth (according to BMI) to one-quarter (according to BIA) of the sample were already obese. This might be indicative of excessive weight already negatively deterring performance on the TGMD-2, however, longitudinal work is needed to understand the directionality of this relationship and examine further the relative contributions body composition plays in FMS performance. Early intervention is needed as over time, the inability to demonstrate competency in these FMS may contribute to lower engagement in play, games, and activities [43] which could consequently contribute to excessive body weight and fat accumulation. In addition, Jones, Okely, Caputi, and Cliff [47] showed that compared to normal weight peers, overweight children tended to have poorer FMS and poorer perceptions of their motor competence (ages 9–11 years) which may also deter future PA engagement, underscoring the importance of understanding critical factors to FMS performance in early childhood.

This study has several limitations that warrant consideration in interpreting the findings. First, this was a cross-sectional investigation, so directionality and causality of the associations cannot be determined. Second, this study used BIA, which is a valid and reliable measure of body composition, but does have limitations including hydration status and muscle fiber composition, which may alter results. Third, the Tanita scale used in the present study does have an age limit of 5 years so we acknowledge this has not been validated in this specific population. However, this scale has been used in previous research in preschool-age children, and does provide a less invasive and cost-effective way to assess body composition in this population in a field setting. Concurrent validity is needed to validate a field-based BIA assessment with a gold-standard measure like DXA and/or skinfold measurements regarding body composition in children under the age of five. Finally, other environmental or individual variables likely contributed to FMS performance and future work should consider additional variables and the combination of process- and product-oriented FMS assessment to better understand FMS competency more holistically in this age group. Further investigation is warranted on the influence of these particular body composition variables and their influence on FMS development and individual skill differences.

## Conclusions

This study found that BMI may be limited in its ability to explain different performances on motor skill assessments. A more nuanced measure of body composition, like BIA, may be more appropriate to examine and understand various types of motor skill performances in children, which are critical for overall health, particularly in reference to locomotor skills. This work found that BIA accounted for more variance in FMS performance than BMI, suggesting this field-based measure may be more appropriate for understanding body composition and its relationship to different types of FMS in children.

## Data Availability

The datasets generated and/or analysed during the current study are not publicly available due to lack of informed consent for data sharing at the time of collection, but are available from the corresponding author on reasonable request.

## Abbreviations

BMI: Body mass index
FMS: Fundamental motor skills
BIA: Bio-electrical impedance analysis
DXA: Dual-energy X-ray absorptiometry
CDC: Centers for Disease Control and Prevention
TGMD-2: Test of Gross Motor Development – 2nd edition
